# Measurement of Liver Iron Concentration by MRI Is Reproducible

**DOI:** 10.1155/2015/294024

**Published:** 2015-03-22

**Authors:** José María Alústiza, José I. Emparanza, Agustín Castiella, Alfonso Casado, Adolfo Garrido, Pablo Aldazábal, Manuel San Vicente, Nerea Garcia, Ana Belén Asensio, Jesús Banales, Emma Salvador, Aranzazu Moyua, Xabier Arozena, Miguel Zarco, Lourdes Jauregui, Ohiana Vicente

**Affiliations:** ^1^Osatek, Donostia Universitary Hospital, P. Dr. Beguiristain 109, 20014 Donostia/San Sebastián, Spain; ^2^Clinical Epidemiology, Donostia Universitary Hospital, P. Dr. Beguiristain 117, 20080 Donostia/San Sebastián, Spain; ^3^Gastroenterology, Mendaro Hospital, Mendarozabal s/n, Mendaro, Spain; ^4^Basque Country University, Avenida Tolosa 54, 20018 Donostia/San Sebastián, Spain; ^5^Biochemical Laboratory, Donostia Universitary Hospital, P. Dr. Beguiristain 117, 20080 Donostia/San Sebastián, Spain; ^6^Experimental Department, Donostia Universitary Hospital, P. Dr. Beguiristain 117, 20080 Donostia/San Sebastián, Spain; ^7^Liver Diseases Unit, Biodonostia Research Institute, P. Dr. Beguiristain s/n, 20014 Donostia/San Sebastián, Spain; ^8^Policlínica Gipúzkoa, Paseo Miramón 174, 20014 Donostia/San Sebastián, Spain; ^9^Radiology, Quirón Donostia Hospital, Alkolea Parkea 7, 20012 Donostia/San Sebastián, Spain; ^10^Onkologikoa, P. Dr. Beguiristain s/n, 20011 Donostia/San Sebastián, Spain

## Abstract

*Purpose*. The objectives were (i) construction of a phantom to reproduce the behavior of iron overload in the liver by MRI and (ii) assessment of the variability of a previously validated method to quantify liver iron concentration between different MRI devices using the phantom and patients. *Materials and Methods*. A phantom reproducing the liver/muscle ratios of two patients with intermediate and high iron overload. Nine patients with different levels of iron overload were studied in 4 multivendor devices and 8 of them were studied twice in the machine where the model was developed. The phantom was analysed in the same equipment and 14 times in the reference machine. *Results*. FeCl_3_ solutions containing 0.3, 0.5, 0.6, and 1.2 mg Fe/mL were chosen to generate the phantom. The average of the intramachine variability for patients was 10% and for the intermachines 8%. For the phantom the intramachine coefficient of variation was always below 0.1 and the average of intermachine variability was 10% for moderate and 5% for high iron overload. *Conclusion*. The phantom reproduces the behavior of patients with moderate or high iron overload. The proposed method of calculating liver iron concentration is reproducible in several different 1.5 T systems.

## 1. Introduction

Measurement of liver iron concentration (LIC) is the best parameter to assess iron deposits in the body. Accordingly, it is a key parameter to guide the clinical management of patients with primary or secondary hemochromatosis, characterized by iron overload. Indeed, an accurate quantitative assessment of iron levels should be obtained before initiating therapy [1, 2]. Although chemical analysis of liver biopsies is the method employed for the analysis of LIC (i.e., gold standard), it is an invasive approach and results vary widely [3–5]. On the other hand, serum markers of iron metabolism such as ferritin and the transferrin saturation index are imprecise for the assessment of iron overload [1, 6].

Magnetic resonance imaging (MRI) is noninvasive and has been shown to provide accurate results compared to the gold standard. It is widely available across the world and several different models for calculating LIC using MRI; both T2 relaxometry [7–9] and signal intensity ratio (SIR) methods [10–13] are being used with successful results. Nevertheless, these MRI-based approaches have not yet been standardized. Our working group has validated an SIR method to estimate LIC. The model strongly correlated with the true LIC (*r* = 0.937) [11].

To standardize an MRI technique, it is necessary to demonstrate the reproducibility of results of both intra- and intermachine. Various recently published clinical guidelines have recommended individual calibration of each MRI machine to quantify LIC [1, 14]. Phantoms are artificial devices that simulate tissues in imaging techniques. The use of phantoms simulating liver with iron overload in MRI to assess the transferability of results and to calibrate different equipment is attractive for ethical, financial, and practical reasons [1, 12, 15–18].

Thus, our objectives were (i) construction of a phantom to reproduce the behavior of iron overload in the liver by MRI and (ii) assessment of the variability of a previously validated method to quantify LIC between different MRI devices using the phantom and patients.

## 2. Materials and Methods

### 2.1. Generation of a New Phantom for the Measurement of Liver Iron Concentration (LIC)

Our goal was to construct a phantom with various different concentrations of an iron solution that gave SIRs similar to those of patients with moderate or high iron overload. The first step was to calculate the mean liver-to-muscle signal intensity ratios of a cohort of 112 patients divided into normal (<36 *μ*mol Fe/g), moderate (36–80 *μ*mol Fe/g), or high iron concentration (>80 *μ*mol Fe/g) [11].

The second step was to test different iron solutions in distilled water using MRI to identify close matches to these average liver-to-muscle ratios of patients with moderate and high iron overload. One tube with only distilled water simulated the non-iron- containing muscle of the patients. Then, ratios were calculated between the signal intensities from each iron-containing tube and that from the distilled water tube.

These tests were conducted in a 1.5 Tesla MRI scan (Gyroscan ACS-NT; Philips, Best, the Netherlands), which was named the “reference machine” because it was the equipment in which our model for LIC quantification was developed. A bottle with 2 litres of diluted CuSO_4_ was placed in the center to give more signal intensity to the whole of the system. The tubes were surrounded by water to avoid susceptibility artifacts in the tube walls. We investigated several different iron solutions such as Lumirem R, iron 3-chloride (FeCl_3_), and ammonium 2-sulphate ((NH_4_)_2_Fe(SO_4_)_2_). First, we assessed Lumirem (Guerbet, France), a ferrous MRI contrast agent with superparamagnetic iron oxides particles (Ferumoxides) previously reported by Ernst et al. [16]. This type of solution was ruled out for lack of stability due to iron precipitation at the bottom of the tubes. Subsequently, we tested FeCl_3_ and (NH_4_)_2_Fe(SO_4_)_2_ solutions [17], which have shown relaxation rates in MRI and that are linearly correlated with the wet-weight iron concentration in human liver. The solutions were acidified with 0.1 M nitric acid to avoid Fe oxidation and thus prevent iron precipitation. Of these, FeCl_3_ was selected because the decrease in the MRI signal showed a better linear correlation with the increase in iron concentration.

12 different solutions of FeCl_3_ with concentrations ranging from 0.05 to 4 mg Fe/mL were prepared in 5 mL tubes. A curve of SI ratios against Fe concentration was created using 12 tubes. The concentrations with SI ratios best mimicking the average liver-to-muscle ratios for intermediate and high iron overload were selected.

### 2.2. Analysis of the Variability of MRI Measurements in the Same Machine (Repeatability) and in Different Machines (Reproducibility) Using Patients

The repeatability of the measurements was evaluated with eight subjects with different LICs (a volunteer with normal iron metabolism, six patients with primary hemochromatosis, and one with posttransfusional iron overload) being analyzed twice in the reference machine. The interval between two measurements was less than 1 week.

To assess the reproducibility, 9 subjects with different LICs were studied in the reference machine and in four additional different units placed in several hospitals of the same city (i.e., General Electric Signa LX (Waukesha, WI) named “A,” General Electric Signa Excite II (Waukesha, WI) named “B,” Siemens Symphony (Erlangen, Germany) named “C,” and a Philips Intera (Best, the Netherlands) named “D”). These subjects were the aforementioned 8 and an additional one with untreated hereditary hemochromatosis. Each patient was studied once in each machine. The interval between the first and the last MRI scan for each patient was always less than 1 week and no patients underwent therapeutic phlebotomy or received iron chelation therapy during this time.

### 2.3. Analysis of the Repeatability and the Reproducibility of MRI Measurements Using the Phantom

The repeatability of the phantom was tested once a week for 14 weeks in the reference machine.

To analyze the reproducibility, the same phantom was studied on the same five 1.5 T systems (previously named as “reference machine,” “A,” “B,” “C,” and “D” machines).

### 2.4. MRI Technique

We scanned the phantom and the human subjects with the two gradient echo (GRE) sequences used in our previously validated method [13] (IW sequence: TR/TE/flip angle = 120 ms/4 ms/20° and T2 sequence: 120 ms/14 ms/20°) with no surface coils. For subjects, the data acquisition was performed in a breath-hold, with 1 NSA, 10 mm thickness, and 10 and 5 slices for IW and T2 sequences, respectively. For the phantom, 5 perpendicular (axial) 7 mm slices were acquired for each tube (gap = 1 mm, FOV = 300 mm, 2 NSA).

### 2.5. Data Analysis

In scans of the subjects, signal intensity was measured with regions of interest (ROI) > 1 cm^2^: 3 in the right lobe of the liver, avoiding vascular structures, and 2 in the paraspinous muscles. For each sequence, the ROIs were always measured in the same slice. Then, for each subject, the liver-to-muscle ratio was obtained with the mean signal intensities of the liver and muscle tissue, and LIC was calculated using the aforementioned equation [11].

For the phantom, the top and bottom axial sections were excluded from the analysis to avoid partial volume effects. In the three central sections, signal intensity was measured in all tubes and using both sequences, with ROIs greater than 0.5 cm^2^ and placed away from the walls. The signal intensity average was obtained for each tube and the signal intensity ratios of each solution to the iron-free tube were calculated. The equation was then used to obtain estimated LIC using the phantom's intermediate and high iron overload data. As described before, we used the ratios of the 0.3 mg Fe/gr and 0.5 mg Fe/gr tubes to represent moderate iron overload with the IW and T2-weighted sequences, respectively, while the 0.6 and 1.2 mg Fe/gr tubes were used for high iron overload.

### 2.6. Statistical Analysis

Appropriate statistics were calculated for each type of data. Coefficients of variation and ranges (minimum and maximum) were calculated for continuous data.

Comparison of the signal intensity ratios for each tube over the 14 days was performed using one-way repeated measures ANOVA.

Agreement between the estimated LIC for both the tubes and the subjects in the four machines tested and the reference machine was assessed using the Bland-Altman method. Diagnostic concordance (no overload, moderate and high iron overload) between the four machines tested and the reference machine was assessed using Cohen's unweighted kappa.

Calibration of each machine against the reference was studied graphically using calibration curves.

The statistical analysis was carried out using the SYSTAT v13 statistical package.

## 3. Results

### 3.1. Generation of a New Phantom with a Range between 62 *μ*mol Fe/g and 180 *μ*mol Fe/g

Patients with intermediate iron overload in liver biopsies have had a liver-to-muscle signal intensity ratio average of 0.95 in the IW sequence and of 0.47 in the T2, while the corresponding liver-to-muscle ratios averages for patients with high iron overload were 0.35 and 0.12, respectively.

**Figure 1:**
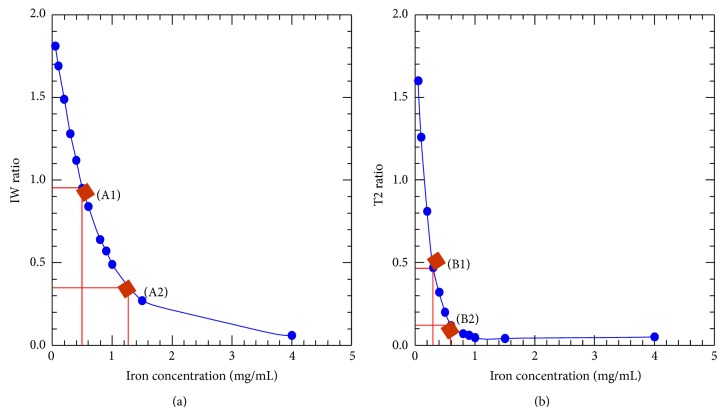
Test of 12 different FeCl_3_ solutions, ranging from 0.05 to 4 mg Fe/mL, to identify signal intensity ratios (SIR) close matches to average liver-to-muscle ratios of patients with moderate or high iron overload. Relationship between the iron concentration and the corresponding SIR in the two sequences of the method. (a) IW sequence (TR/TE/Flip 120/4/20°); (b) T2 sequence (TR/TE/Flip 120/14/20°). SIR was calculated between the signal intensities from each FeCl_3_ solution and that from distilled water, without any iron. In both sequences SIR decreases with increasing iron concentration and it falls more steeply in T2 sequence, as occurs in clinical measurements. It is necessary to have one solution with specific concentration of FeCl_3_ for each sequence and for each level of iron overload. For intermediate iron overload, the solution containing 0.5 mg Fe/mL (A1) gave the required IW signal intensity ratio (0.95) and the one with 0.3 mg Fe/mL (B1) gave the required T2 signal intensity ratio (0.47). For the high iron overload, 1.2 mg Fe/mL (A2) and 0.6 mg Fe/mL (B2) were necessary to obtain the desired IW and T2 ratios (0.35 and 0.6, resp.).

**Figure 2:**
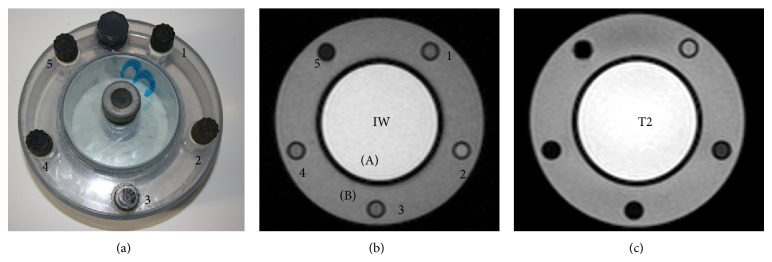
Phantom with different iron (III) chloride solutions and MRI in IW (TR/TE/Flip angle = 120/4/20°) and T2 (120/14/20°) sequences. (a) Photograph of the first prototype. (b) MRI in IW sequence. (c) MRI in T2 sequence. 1. Fe-free solution. 2. Solution of 0.3 mg Fe/mL. 3. Solution of 0.5 mg Fe/mL. 4. Solution of 0.6 mg Fe/mL. 5. Solution of 1.2 mg Fe/mL. (A) CuSO_4_ solution. (B) Water.

Figure 1 illustrates the relationship between the signal intensity ratio in 12 tubes with different FeCl_3_ concentrations ranging from 0.05 to 4 mg Fe/mL for the two sequences. In both cases, signal intensity inversely correlates with the iron concentration, but the signal falls more steeply in the T2 sequence, as occurs in clinical measurements [11]. Two different tubes were necessary to obtain the SI ratios of each level of iron overload. For intermediate iron overload, the solution containing 0.5 mg Fe/mL (A1) gave the required IW signal intensity ratio (0.95) and 0.3 mg Fe/mL (B1) the required T2 signal intensity ratio (0.47). For high iron overload, 1.2 mg Fe/mL (A2) and 0.6 mg Fe/mL (B2) were necessary to obtain the desired IW and T2 ratios (0.35 and 0.6 mg Fe/mL, resp.). Based on this data, the phantom was constructed using one tube without iron and each of the four different FeCl_3_ solutions (Figure 2).

**Figure 3:**
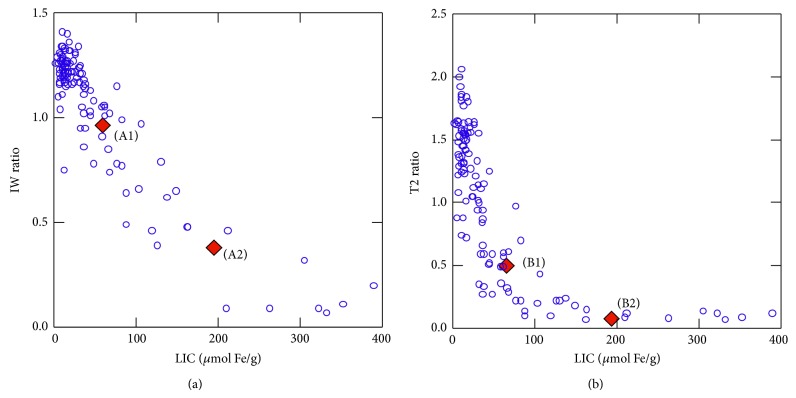
Relationship between liver-to-muscle signal intensity ratio (SIR) and liver iron concentration (LIC) for 112 patients. SIRs of the phantom for moderate (A1-B1) and for high iron overload (A2-B2) in the two sequences of the method have also been included in the graph. (a) IW sequence (TR/TE/Flip 120/4/20°); (b) T2 sequence (TR/TE/Flip 120/14/20°). The values A1 and B1 correspond to the same LIC value in each of the two sequences: 62 *μ*mol Fe/g and they maintain the same correlation SIR/LIC as patients in the two sequences. The same applies to A2 and B2 values for high iron overload, with a value of LIC of *μ*mol Fe/g in both sequences.

A subject with IW and T2 ratios corresponding to intermediate iron overload would be predicted to have an LIC of 62 *μ*mol Fe/g by our equation. On the other hand, a person with high iron overload ratios would be estimated to have an LIC of 180 *μ*mol Fe/g. These values are very similar to those obtained in our groups of real patients with moderate (51 *μ*mol Fe/g) and high (187 *μ*mol Fe/g) iron overload (Figure 3).

### 3.2. The Patients Show Good Reproducibility of Measurements Intra- and Intermachines

Of the 9 patients studied, 3 have a normal value of LIC (<36 *μ*mol Fe/g), 2 have a moderate iron overload (37–80 *μ*mol Fe/g), and 4 have high iron overload (>80 *μ*mol Fe/g).

**Figure 4:**
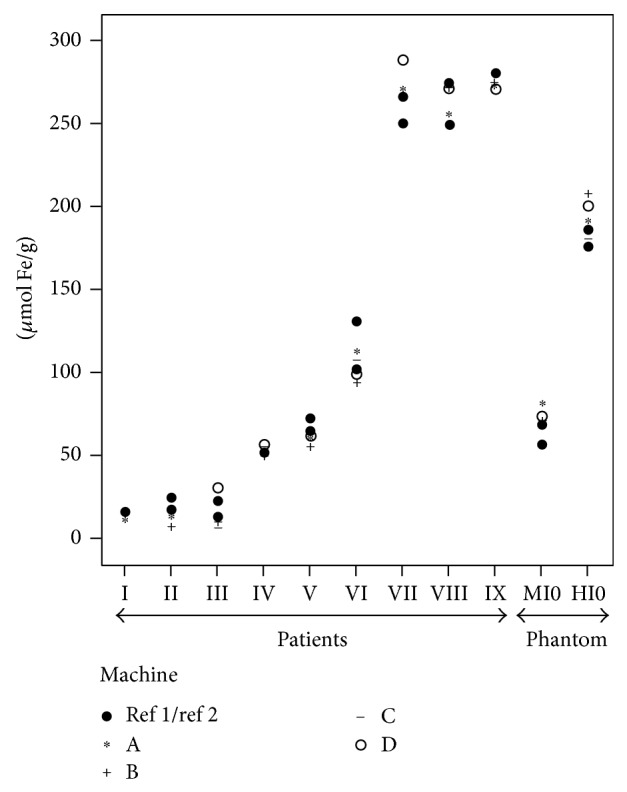
Variability of estimates by MRI of liver iron concentration (LIC) in 9 patients and in the phantom. I–IX: real patients with different values of LIC. MIO: moderate iron overload in the phantom. HIO: high iron overload in the phantom. Black rounds: measurements on the reference machine. “∗”: measurements on machine A. “+”: measurements on machine B. “−”: measurements on machine C. “∘”: measurements on machine “D”. High reproducibility can be observed, intra- and intermachines, for every patient. Phantom behavior is very similar to the patients.

Between the 8 patients studied twice in the reference machine, the average of intramachine variability was 10% (2-22) (Figure 4). There were no clinically relevant differences between the two measurements.

The variability of all machines analyzed with respect to the reference machine was low, ranging from 0 to 28%, with a mean difference of 8% in patients with iron overload (Figure 4).

**Figure 5:**
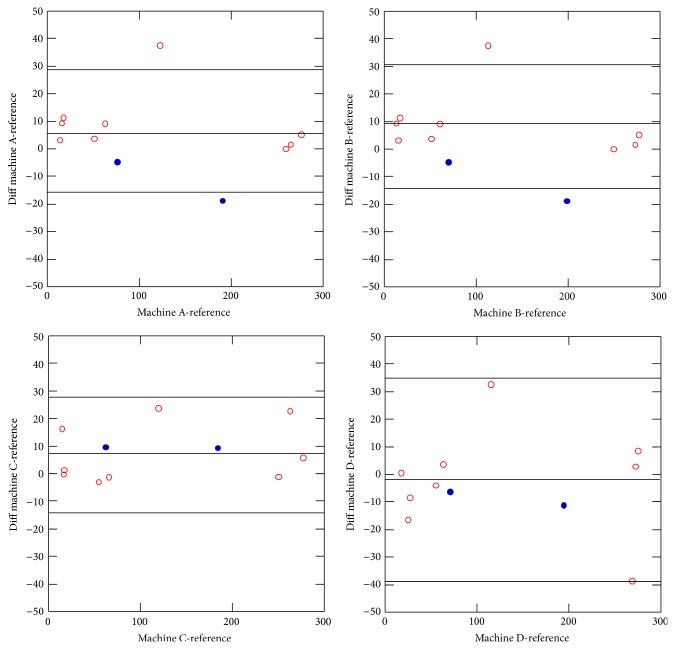
Bland-Altman plots for each machine (machines A, B, C, and D) and the reference machine. The Bland-Altman plot shows an agreement within the limits of clinical usefulness, with the mean of differences (bias) always being less than 20%. Specifically, the bias ranged from −3.4 to 7.4 *μ*mol Fe/g, which can be considered negligible values.

The Bland-Altman plot (Figure 5) displays an agreement within the limits of clinical usefulness, with the mean of differences always being less than 20%. Specifically, the differences ranged from −3.4 to 7.4 *μ*mol Fe/g, which can be considered negligible values.

All the subjects without iron overload (<36 *μ*mol Fe/g) were classified with the other four machines (A–D). Importantly, none of the patients with moderate (37–80 *μ*mol Fe/g) or high (>80 *μ*mol Fe/g) iron overload were classified as not having iron overload by any of the four machines tested. In addition, the patients were all classified in the correct iron overload group by the tested machines. Cohen's kappa was 1, reflecting the almost perfect agreement in diagnosing the severity of iron overload.

### 3.3. The Phantom Has Similar Behavior to Patients Intra- and Intermachines

The variability in the 14 measurements of the phantom made in the reference machine was also very low, with a coefficient of variation between 0.02 and 0.09.

All the estimated values for moderate iron overload were between 56 and 68 *μ*mol Fe/g and for high iron overload between 176.1 and 186.2 *μ*mol Fe/g. (Figure 3).

The variability of the measurements in the phantom of all the analyzed machines with respect to the reference machine was also very low. It was 10% (1-19) for moderate iron overload and 5% (1-10) to high overload (Figure 4).

## 4. Discussion

In this study we have generated a new phantom ranking between 62 *μ*mol Fe/g and 180 *μ*mol Fe/g. We show that patients have good reproducibility of measurements and that the phantom has similar intra- and intermachines.

We designed an MRI phantom which accurately reproduces the LIC of two typical groups of patients, namely, those with moderate and high iron overload, which is stable over time. Other studies have used phantoms based on various different components including agarose, ferritin, manganese, and Lumirem [15–18]. Clearly it is not feasible to construct an artificial phantom with only one component which exactly reproduces the behavior of the liver with various different iron concentrations in MRI, as the liver is a highly complex biological structure. The aqueous solution of ferric chloride and water without iron that we have used in this study has different T1 and T2 values compared to liver tissue with iron overload or to the muscle.

On the other hand, in constructing a phantom, the most important issue is to employ a properly studied and validated gold standard. We have based our design on the data of a cohort of 112 patients, analyzed using the same MRI machine and who also have an LIC quantified using chemical measurements in liver biopsies, provided that, using tubes with different FeCl_3_ solutions, we were able to reproduce accurately the behavior seen in patients with different levels of iron overload. In this regard, as stated in Figure 1, the change in the signal from the tubes with the increase in iron concentration was very similar to that from the patients with different values of LIC in the two gradient echo sequences used in the model. In addition, in both sequences, the theoretical LICs of patients as mimicked by the phantom were perfectly correlated with the gold standard (Figure 4).

To summarize, there cannot be a single solution of FeCl_3_ that gives the same SIR as the liver in different sequences. Therefore it is necessary to have a specific tube for each sequence and for each level of iron overload. Hence, the phantom consists of four tubes with different FeCl_3_ concentrations and one tube without iron.

This study shows that the SIR model for the estimation of LIC using MRI designed by our research group [11] is reproducible in 1.5 T machines of various different companies. In particular, none of the patients without iron overload was diagnosed as having iron overload by results from any of the machines. Furthermore, the patients with iron overload were also correctly classified in all cases.

SIR methods to quantify LIC by MRI are more available than T2 relaxometry methods and they are widely used in clinical practice. However, very few have analyzed the reproducibility of the measurements in different machines. So far there are no published studies that have compared the measures of so many patients and machines as this study [15]. The variability we found is very similar intra- and intermachines (8–10%) and it is better than that observed and implicitly accepted in clinical practice in the measurement of LIC by biopsy, in which results vary by 19% in healthy liver and 40% in cirrhotic liver tissue [3–5].

The phantom has shown similar behavior to the patients with moderate and high iron overload. Indeed, an important future use of the phantom, freely available on request, will be to undertake calibrations in more centres. Such data, in a multicentre study, would ensure consistency between the calculated values of LIC from different machines. This will be a necessary step towards the standardization of the technique that will optimize their use in the differential diagnosis of disorders of iron metabolism.

This study has some limitations. It is based on only nine subjects and four machines. However, it is difficult to identify patients and healthy individuals willing to undergo repeated measurements in different hospitals in a short period of time. Iron concentrations were not confirmed by biopsy. On the other hand, we have previously demonstrated that the method provides accurate estimates of LIC; for this reason, biopsies are no longer taken to measure liver iron concentration in our hospital. All results of this study are only valid for the used quantification method, previously designed and validated by our team.

In conclusion, this study demonstrates that the proposed signal-to-intensity ratio method to calculate LIC by MRI is reproducible in several different 1.5 T systems (from different companies) and also that the behavior of the constructed phantom reproduces the behavior of patients with moderate or high iron overload.
